# Bentho-Pelagic Divergence of Cichlid Feeding Architecture Was Prodigious and Consistent during Multiple Adaptive Radiations within African Rift-Lakes

**DOI:** 10.1371/journal.pone.0009551

**Published:** 2010-03-08

**Authors:** W. James Cooper, Kevin Parsons, Alyssa McIntyre, Brittany Kern, Alana McGee-Moore, R. Craig Albertson

**Affiliations:** 1 Department of Biology, Syracuse University, Syracuse, New York, United States of America; 2 School of Dental Medicine, University of Connecticut, Storrs, Connecticut, United States of America; University of Hull, United Kingdom

## Abstract

**Background:**

How particular changes in functional morphology can repeatedly promote ecological diversification is an active area of evolutionary investigation. The African rift-lake cichlids offer a calibrated time series of the most dramatic adaptive radiations of vertebrate trophic morphology yet described, and the replicate nature of these events provides a unique opportunity to test whether common changes in functional morphology have repeatedly facilitated their ecological success.

**Methodology/Principal Findings:**

Specimens from 87 genera of cichlid fishes endemic to Lakes Tanganyka, Malawi and Victoria were dissected in order to examine the functional morphology of cichlid feeding. We quantified shape using geometric morphometrics and compared patterns of morphological diversity using a series of analytical tests. The primary axes of divergence were conserved among all three radiations, and the most prevalent changes involved the size of the preorbital region of the skull. Even the fishes from the youngest of these lakes (Victoria), which exhibit the lowest amount of skull shape disparity, have undergone extensive preorbital evolution relative to other craniofacial traits. Such changes have large effects on feeding biomechanics, and can promote expansion into a wide array of niches along a bentho-pelagic ecomorphological axis.

**Conclusions/Significance:**

Here we show that specific changes in trophic anatomy have evolved repeatedly in the African rift lakes, and our results suggest that simple morphological alterations that have large ecological consequences are likely to constitute critical components of adaptive radiations in functional morphology. Such shifts may precede more complex shape changes as lineages diversify into unoccupied niches. The data presented here, combined with observations of other fish lineages, suggest that the preorbital region represents an evolutionary module that can respond quickly to natural selection when fishes colonize new lakes. Characterizing the changes in cichlid trophic morphology that have contributed to their extraordinary adaptive radiations has broad evolutionary implications, and such studies are necessary for directing future investigations into the proximate mechanisms that have shaped these spectacular phenomena.

## Introduction

The cichlid fishes (Perciformes, Cichlidae) that inhabit the major lakes of East Africa's rift valley have produced the most remarkable adaptive radiations in vertebrate feeding morphology ever described. In a short amount of geological time (no more than 16 MY), Lakes Tanganyika, Malawi and Victoria have fostered the explosive evolution of hundreds of cichlid species [1]–[11]. The present trophic diversity of these fishes, which constitute only a portion of the Cichlidae, is comparable to the aggregate of that present in multiple perciform fish families of considerably greater ages. A large body of scientific work has focused on determining the processes that have generated these incredible evolutionary phenomena [e.g.], [ 7], [12], [13]–[15], but so far there is no widely accepted explanation or set of explanations that can entirely account for the rapid diversification of these fishes.

An important step towards understanding such processes is the accurate quantification of the patterns they have produced. Since the cichlids in each of these lakes appear to have undergone similar types of morphological diversification [11] (but see [16]), and since the ages of the lakes span at least two orders of magnitude [17], these groups of fishes approximate replicate radiations at different stages of expansion [11]. This situation presents an unparalleled opportunity to examine well-spaced chronological snapshots of evolutionary divergence in the functional morphology of cichlid feeding. The most important goals of this study were to: 1) determine if there have been consistent types of craniofacial change during the adaptive radiations of trophic morphology that have occurred among the East-African rift-lake cichlids; 2) to quantify and describe these changes in order to gain a more explicit understanding of the patterns that underlie these important radiations; and 3) to determine if any specific anatomical changes have generated particularly strong expansions of ecomorphological diversity. The achievement of these goals will provide an important foundation for future investigations into the factors that have permitted and directed these evolutionary events. In addition to improving our understanding of how cichlids have diversified in these lakes, establishing whether particular morphological changes of functional importance have repeatedly led to similar patterns of ecological divergence could permit a better understanding of the nature of adaptive radiations in other groups of fishes as well.

## Methods

Except where noted, most of the analytical software used during this study (i.e., the programs CoordGen, CVAGen6o, DisparityBox6, PCAGen, and SpaceAngle) are part of the Integrated Morphometrics Programs (IMP) series created by David Sheets, and compiled stand-alone versions that run in Windows are freely available at http://www3.canisius.edu/~sheets/morphsoft.html.

### Specimens and Data Collection

Cichlid genera are typically defined by morphological differences, whereas species differences are generally based on male nuptial coloration [18], [19]. Our sampling was therefore directed at obtaining specimens from a large percentage of the cichlid genera endemic to each of the three major East-African rift lakes. We examined 78.8% of the genera that are endemic to all three lakes, with the following percentages from each of the individual lakes: Tanganyika (74.5%), Malawi (88.5%), and Victoria (57.1%). Specimens were provided by the American Museum of Natural History, the Belgian Royal Museum for Central Africa, Cornell University's Museum of Vertebrates, Harvard University's Museum of Comparative Zoology, and the University of Michigan's Museum of Zoology (Table S1). A small number of specimens were also obtained through the live fish trade. Taxonomy follows the July 2, 2009 update of the California Academy of Sciences' electronic version of the Catalog of Fishes (http://research.calacademy.org/ichthyology/catalog/fishcatsearch.html) [20].

**Figure 1:**
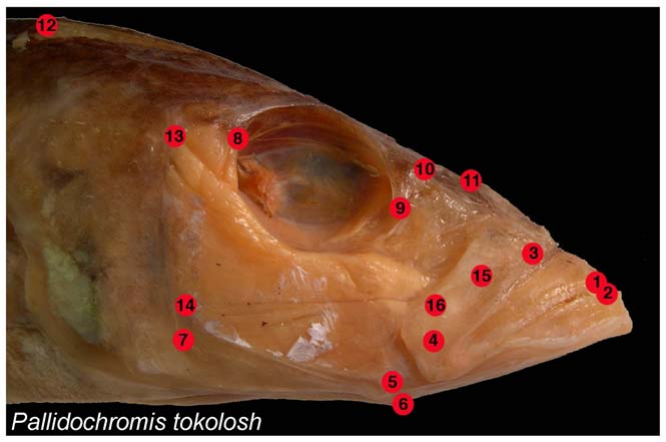
Anatomical landmarks examined. 1 = Tip of the anterior-most tooth on the premaxilla; 2 = Tip of the anterior-most tooth on the dentary; 3 = Maxillary-palatine joint (upper rotation point of the maxilla); 4 = Maxillary-articular joint (lower point of rotation of the maxilla); 5 = Articular-quadrate joint (lower jaw joint); 6 = Insertion of the interopercular ligament on the articular (point at which mouth opening forces are applied); 7 = Posterio-ventral corner of the preopercular; 8 = Most posterio-ventral point of the eye socket; 9 = The most anterio-ventral point of the eye socket; 10 = Joint between the nasal bone and the neurocranium; 11 = Posterior tip of the ascending process of the premaxilla; 12 = Dorsal-most tip of the supraoccipital crest on the neurocranium; 13 = Most dorsal point on the origin of the A1 division of the *adductor mandibulae* jaw closing muscle on the preopercular; 14 = Most dorsal point on the origin of the A12 division of the *adductor mandibulae* jaw closing muscle on the preopercular; 15 = Insertion of the A1 division of the *adductor mandibulae* on the maxilla; 16 = Insertion of the A2 division of the *adductor mandibulae* on the articular process.

Dissections, specimen photography, the establishment of spatial coordinates for anatomical landmarks, and Procrustes transformations of landmark data largely follow Cooper and Westneat [21]. Dissections were performed on cichlid heads in order to expose anatomical landmarks of importance for oral jaw functioning (Figure 1). The right side of the head was dissected in most cases, but data from both sides were taken from the laterally asymmetrical heads of several scale-eating species from Lake Tanganyika. Cleared and stained specimens were carefully examined in order to determine those landmarks associated with the jaw adductor muscles. Heads were photographed in lateral view with a scale bar, and with their mouths closed and their operculae and hyoid arches adducted. In most cases more than one specimen of each species was obtained, and in almost every case adult fishes were examined. Digital photographs were taken using an Olympus SP-570 digital camera. Sixteen (16) anatomical landmarks (Figure 1) were plotted on each image using the software program tpsDig [22].

### Initial Transformations and Primary Shape Analyses

Images of individual specimens can vary due to differences in size, orientation and translation. To remove these effects a Procrustes sumperimposition of the landmark data was performed using the program CoordGen. Procrustes transformations superimpose the landmarks of all specimens as much as possible without distorting their shape, and scale the landmark clusters of each specimen to the same centroid size. The mean coordinate configuration of each species was then calculated from the Procrustes transformed landmark coordinates using Excel (Microsoft, Corp.). This calculation of a mean shape allows for a more accurate characterization of the morphology of a given species than can usually be achieved by examining a single specimen.

The mean Procrustes coordinate configurations for every species were combined in a single data matrix, and a Procrustes transformation was used once again to remove size and orientation effects. This combined dataset was then partitioned so as to create separate matrices for the data derived from each lake. The datasets analyzed in this study were: all of the species from all lakes (All Lakes), species from Lake Tanganyika (LT), species from Lake Malawi (LM), and species from Lake Victoria (LV).

To assess the primary patterns of morphological variation present in each lake we used a principal components analysis (PCA). PCAs of partial warp (PW) scores derived from the Procrustes transformed data were used to individually analyze all four datasets. Deformation grids were used as aids for determining the type of morphological shape variation described by individual PC axes, and scree plots of eigenvalues were used to depict the partitioning of shape variation among the PC axes derived from each group of data. The program PCAGen was used both to perform these PCAs and to generate deformation grids. A canonical variance analysis (CVA) was also performed using the program CVAGen6o in order to determine the number of significant canonical variate (CV) axes (if any) that could distinguish the sets of data for the individual lakes.

PCAs of combined datasets can artificially force the major axes of shape variation present in the more variable datasets through the data derived from the less variable dataset or sets. Combining groups of data in order to perform a common PCA without first determining if their major axes of shape variation are similar can therefore generate misleading results in regard to whether particular types of shape variation (PC axes) are of similar importance to the component groups [16]. It must also be considered that although CVA will detect significant differences between sets of data, in the case of shape comparisons, this method is blind as to whether such differences are due to the presence of different types of shape variation (e.g., differently oriented PC axes), the degree of morphological diversity that each groups exhibits, or a combination of these factors. We therefore tested for both differences in morphological diversity (disparity), and for differences in the types of shape variation present in each lake (i.e., differences in the orientation of their shape spaces), in order to isolate the specific causes of any existing shape dissimilarities, and to assist our interpretation of the results of the PCA of the All Lakes dataset.

### Examining Patterns of Morphological Integration

Morphological integration occurs when there is “cohesion among traits”, and when the integration among anatomical landmarks is high their positions will covary strongly [23], [24]. Such convariation could be favored by selection or enforced by developmental constraints, but in either case, whenever the positions of anatomical landmarks are linked, the morphological structures being examined have a restricted freedom to vary in shape. When highly integrated morphometric data are analyzed using PCA, the high levels of positional covariation will skew the distribution of shape variation among the PC axes such that most of the variation will be explained by only a few axes [24]–[27]. This would be reflected by the existence of one or a few axes that explain a high amount of variation followed by a ‘distinct’ drop in explanatory power in subsequent PCs.

Patterns of integration are usually investigated using PCA or similar methods [23], and PCA is a useful tool for searching for patterns of morphological integration using landmark data [28], [29]. We used a Chi-squared test to determine if and when there was a strong drop (beginning with the first PC axis and proceeding onward) in the explanatory power (eigenvalues) of the PC axes. This method determines whether the total shape variation in a dataset is spread out among many PC axes (low level of integration) or concentrated within a small number of the initial PC axes (higher level of integration). The process is sequential, and begins with pairwise comparisons of the first two axes and continues (PC1 vs. PC2, PC2 vs. PC3, etc.) until it is determined that the amounts of variation explained by a pair of components are not significantly different (Chi-square statistic; alpha = 0.05). We used this approach to compare patterns of morphological integration among the three radiations of rift-lake cichlids. For a detailed explanation of the method see p. 211–254 in Morrison [30]. The program PCAGen was used to generate scree plots and to determine the number of distinct PC axes in each dataset.

### Comparisons of Morphological Disparity

The morphological disparity exhibited by the cichlids from each lake was calculated using the method established by Foote [31], in which:
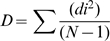


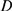
 denotes the “Foote disparity” value, 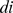 is the distance from the centroid of the entire group (the centroid of the mean shape of all the species in a dataset) to the centroid of the Procustes mean shape of each individual species in that group, and 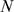 is the total number of species in that dataset. Bootstrapping (2500 sets) was used to establish 95% confidence intervals (CI) for the disparity values calculated for each lake.

For pairwise comparisons of the disparity exhibited by the fishes from two different lakes, a permutation procedure (1000 runs) was used to calculate the 95% CI of a generated disparity difference. The Procrustes mean landmark configurations for individual species were randomly assigned to two groups equal in size to the two original groups (i.e., the number of species sampled from each of the two lakes being compared), the Foote disparity values were then calculated for each of these new, randomly compiled groups, the between groups disparity difference was recorded, and this procedure was repeated 1,000 times in order to generate a distribution of random disparity differences. This distribution was used to calculate 95% confidence intervals for the mean random disparity difference. If the actual disparity difference between the two original groups was greater then the upper bound of this interval, then the difference in morphological disparity between the samples derived from the two lakes being compared was considered to be significant. The program DisparityBox6 was used to perform these calculations.

### Comparing the Trajectories of Major Axes of Divergence

Adaptive radiations may differ in overall diversity, but still share important morphological trajectories among their respective axes of divergence. We therefore conducted pairwise tests to determine whether shape space orientations, as defined by PC axes, were different among these cichlid radiations. Specifically, we used the program SpaceAngle to determine if the observed angle between two shape spaces differed from those calculated from random sub-divisions of either dataset. A bootstrapping procedure (2500 sets) was used to define the 95% CI for the angles calculated from re-sampling each of the two original datasets being compared. If the observed angle fell within either of the two 95% CIs, then the orientations of the two original shape spaces were not considered to be significantly different.

The orientations of the first PC axes derived from each of the datasets were compared directly, but axes subsequent to PC1 could not be examined individually. All analyses that involved multiple axes determined whether the alignments of planes (when only 2 PC axes were examined) or multi-dimensional hyperplanes (“flat” surfaces of >2 dimensions embedded in larger dimensional spaces) were significantly different. When bootstrapping any two of the original sets of data, the sample sizes of the bootstrap sets produced from the larger of the two were the same as the sample sizes of both original datasets. Re-sampling the smaller of the original two datasets created two bootstrap sets of the same size as the original.

Shared axes of divergence may still differ among groups with respect to the average shape they are associated with, and with the variation in PC scores on a particular axis. Therefore, for those PCs that were common between all datasets, a one-way analysis of variance (ANOVA) was used to determine if the mean PC scores for a specific PC axis differed between groups. A Levene's test was used to determine if the variance in these scores were significantly different when comparing the data from more than two lakes, and pairwise comparisons of score variance were performed using an F-test. When the ANOVA assumption of equal variances among samples was violated, non-parametric Kruskal-Wallis tests for equal median values were also used. Due to the performance of multiple comparisons using the same datasets, a Tukey's HSD (honestly significant difference) test with a 95% CI was used to perform a post-hoc test of significance subsequent to each ANOVA in order to reduce the chance of committing Type I errors. When needed, a sequential Bonferroni correction [32] was used to adjust the alpha level used when performing multiple F-tests of equal variance (original alpha = 0.05). The one-way ANOVAs and Tukey's HSD test were performed using XLSTAT (Addinsoft, USA). The Levene's tests, F-tests and Kruskal-Wallis tests were performed using Systat 12 (Systat Software, Inc.).

## Results

### Comparisons of Morphological Diversity and Disparity

**Figure 2:**
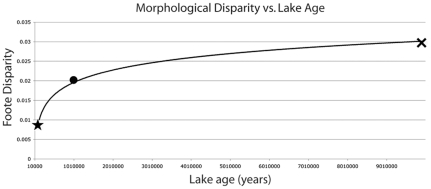
Morphological disparity relative to lake age. (**X** = LT species; • = LM species; ★ = LV species). The trendline depicted does not denote a significant relationship between a lake's age and the morphological disparity of its cichlids.

The overall patterns of the skull shapes present in each lake were statistically different. There were two significant CV axes (Axis 1 λ = 0.0507 χ^2^ = 245.9699 df = 56 p<0.001; Axis 2 λ = 0.2765 χ^2^ = 106.0607 df = 27 p<0.001), both of which distinguished between all three groups. All between lakes comparisons of cichlid head shape disparity revealed significant differences, with relative disparity rankings as follows: LT>LM>LV (Table 1; Figure 2). The low sample size (three lakes) does not permit a statistical analysis of the relationship between the age of a lake and the head shape diversity of its constituent cichlids, but the pattern observed is consistent with the existence of a positive relationship between these two variables (Figure 2).

**Table 1:** Comparative disparity of cichlid head morphology.

Lake	Foote disparity		95% confidence interval	Standard Error
Tanganyika	0.030		0.025–0.033	0.002
Malawi	0.020		0.016–0.023	0.002
Victoria	0.008		0.004–0.011	0.002

Comparison	Observed disparity difference		95% upper bound of the permutation generated difference	Number of permutations in which the magnitude of the observed difference was exceeded
LT vs. LM	0.009*	>	0.006	1
LT vs. LV	0.021*	>	0.011	0
LM vs. LV	0.012*	>	0.008	4

95% CIs and standard errors were derived from 2500 bootstrap sets. Between lakes comparisons were identified as significantly different when the observed difference was >the 95% upper bound of the permutation generated difference (1000 permutation runs).

*denotes a significant difference.

### Shared Major Axes of Shape Diversification

**Figure 3 pone-0009551-g003:**
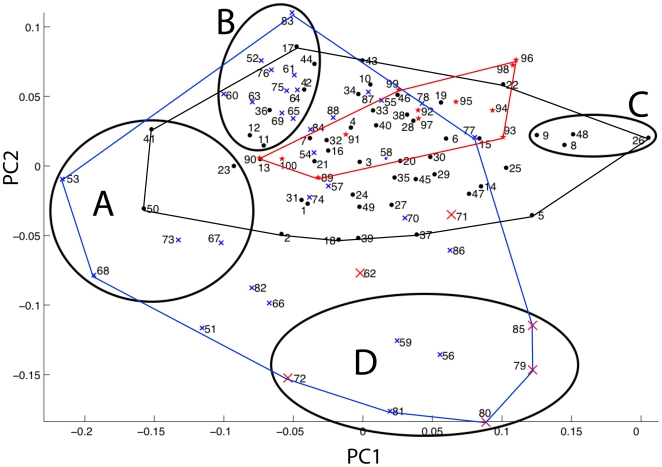
PC score plot of the All Lakes data with ecomorphological groupings. **X** = LT species (blue); Tropheini from Lake Tanganyika = large **X** (red); • = LM species (black); ★ = LV species (red); A = long jawed predators; B = pelagic fishes with large eyes placed near gracile, protrusile jaws; C = Hard biting fishes from Lake Malawi. D = Hard biting fishes from Lake Tanganyika. The key to the species can be found in Table S2.

The score plot generated from the PCA of the All Lakes dataset, and between lakes comparisons of PC axis orientations, indicate that major axes of shape variation are common to all three radiations (Table 2, Figure 3). There are strongly overlapping distributions of the three groups on the first two PC axes (Figure 3), which respectively account for 23.7% and 14.4% of the total shape variation. Especially similar were the trajectories of divergence from LM and LV, where stepwise comparisons of sequentially larger sets of PC axes showed no significant differences even when all PC axes were compared.

**Table 2:** Comparative shape space orientations.

Comparison (PC1)	Observed angle	Range of the 95th% CI	Range of the 95th% CI
(LM+LV) vs. LT	5.89	(LM+LV): 0.97–10.87	LT: 0.96–11.84
(LT+LV) vs. LM	2.38	(LT+LV): 0.99–4.65	LM: 0.99–5.44
(LT+LM) vs. LV	9.72	(LT+LM): 0.96–8.43	LV: 0.90–18.50
LT vs. LM	8.56	LT: 0.95–12.24	LM: 0.99–5.91
LT vs. LV	15.25	LT: 0.96–12.13	LV: 0.89–19.21
LM vs. LV	9.27	LM: 0.99–5.38	LV: 0.90–18.56

Comparison (PC1+PC2)	Observed angle	Range of the 95th% CI	Range of the 95th% CI
(LM+LV) vs. LT	64.32	(LM+LV): 1.39–85.23	LT: 1.00–86.71
(LT+LV) vs. LM	32.34	(LT+LV): 1.89–63.23	LM: 1.00–85.78
(LT+LM) vs. LV	23.45	(LT+LM): 1.77–54.65	LV: 1.28–57.41
LT vs. LM	58.08	LT: 1.00–85.84	LM: 1.00–87.04
LT vs. LV	74.46	LT: 1.00–86.51	LV: 1.22–61.55
LM vs. LV	33.63	LM: 1.00–87.32	LV: 1.27–57.88

Bootstrap confidence intervals for the observed angles between shape spaces were calculated by resampling the data from both groups. Orientations were not considered significantly different if the observed angle fell within either CI. None of the orientations for the axes and planes compared above were significantly different.

The datasets from all three lakes had similar levels of variation in their PC1 scores (Table 3). Only the LM and LV datasets exhibited similar variation in their PC2 scores, while the variance of the LT PC2 scores was significantly different from those of both the LV and LM datasets (Table 3). Kruskal-Wallis tests for equal medians were therefore performed for both the LT vs. LV and the LT vs. LM comparisons of median PC2 scores (Table 4).

**Table 3:** Tests for homogeneity of variance.

Test	Comparison	Test statistic	p-value
**Levene's test** (multiple samples)	All three lakes (PC1)	0.210	0.811
	All three lakes (PC2)	3.928	0.023*
**F-test** (PC1)	LT (0.004) vs. LM (0.005)	F-ratio = 1.340	0.357
	LT vs. LV (0.004)	F-ratio = 1.055	0.994
	LM vs. LV	F-ratio = 1.413	0.574
**F-test** (PC2)	LT (0.006) vs. LM (0.003)	F-ratio = 0.471	0.014*
	LT vs. LV (0.001)	F-ratio = 4.137	0.021*
	LM vs. LV	F-ratio = 1.950	0.254

The PC score variance is given once in parentheses for each individual lake dataset.

*denotes a significant difference.

**Table 4:** Comparisons of mean and median PC scores.

Test	Comparison	F-ratio	p-value			
**One-way ANOVA (PC1)**	All Lakes	0.520	0.596			

	Comparison	Difference	Standardized difference	Critical value	Pr > Diff	Significant
**Tukey's HSD (PC1)** (critical value = 3.367)	LT (−0.023) vs LM (0.010)	<0.001	0.308	2.381	0.949	No
	LT vs LV (0.044)	0.001	0.798	2.381	0.705	No
	LM vs LV	0.001	1.019	2.381	0.567	No

	Comparison	F-ratio	p-value			
**One-way ANOVA (PC2)**	All Lakes	0.004	0.996			

	Comparison	Difference	Standardized difference	Critical value	Pr > Diff	Significant
**Tukey's HSD (PC2)** (critical value = 3.367)	LT (−0.026) vs LM (0.020)	<0.001	0.013	2.381	1.000	No
	LT vs LV (−0.012)	<0.001	0.074	2.381	0.997	No
	LM vs LV	<0.001	0.084	2.381	0.996	No

	Comparison	Mann-Whitney U Test Statistic	p-value	Chi-square approximation		
**Kruskal-Wallis (PC2)**	LT vs LM	987.0	0.595	0.283		
	LT vs LV	180.0	0.564	0.332		

Mean PC scores are given once in parentheses for each individual lake dataset. ANOVA and Tukey's HSD tests examined mean PC scores. Non-parametric Kruskal-Wallis one-way ANOVAs examined median PC scores.

None of the datasets hade significantly different mean PC1 scores, or significantly different mean or median PC2 scores (Table 4). One-way ANOVAs performed on the PC2 scores from all lakes (analyzed together) indicated that there were no significant differences in PC2 scores (Table 4; although the assumption of equal variation was not upheld for all comparisons). The results of pairwise one-way ANOVAs (Table 4) also indicated that there were no significant differences in mean PC2 scores for any of the between lakes comparisons (LT vs. LM: F ratio = <0.001, p = 0.989; LT vs. LV: F ratio = 0.006, p = 0.940; LM vs. LV: F ration = <0.001, p = 0.998), and the results of the Kruskal-Wallis tests indicated that the median PC2 scores were not significantly different for the LT vs. LV or LT vs. LM comparisons (Table 4).

**Figure 4 pone-0009551-g004:**
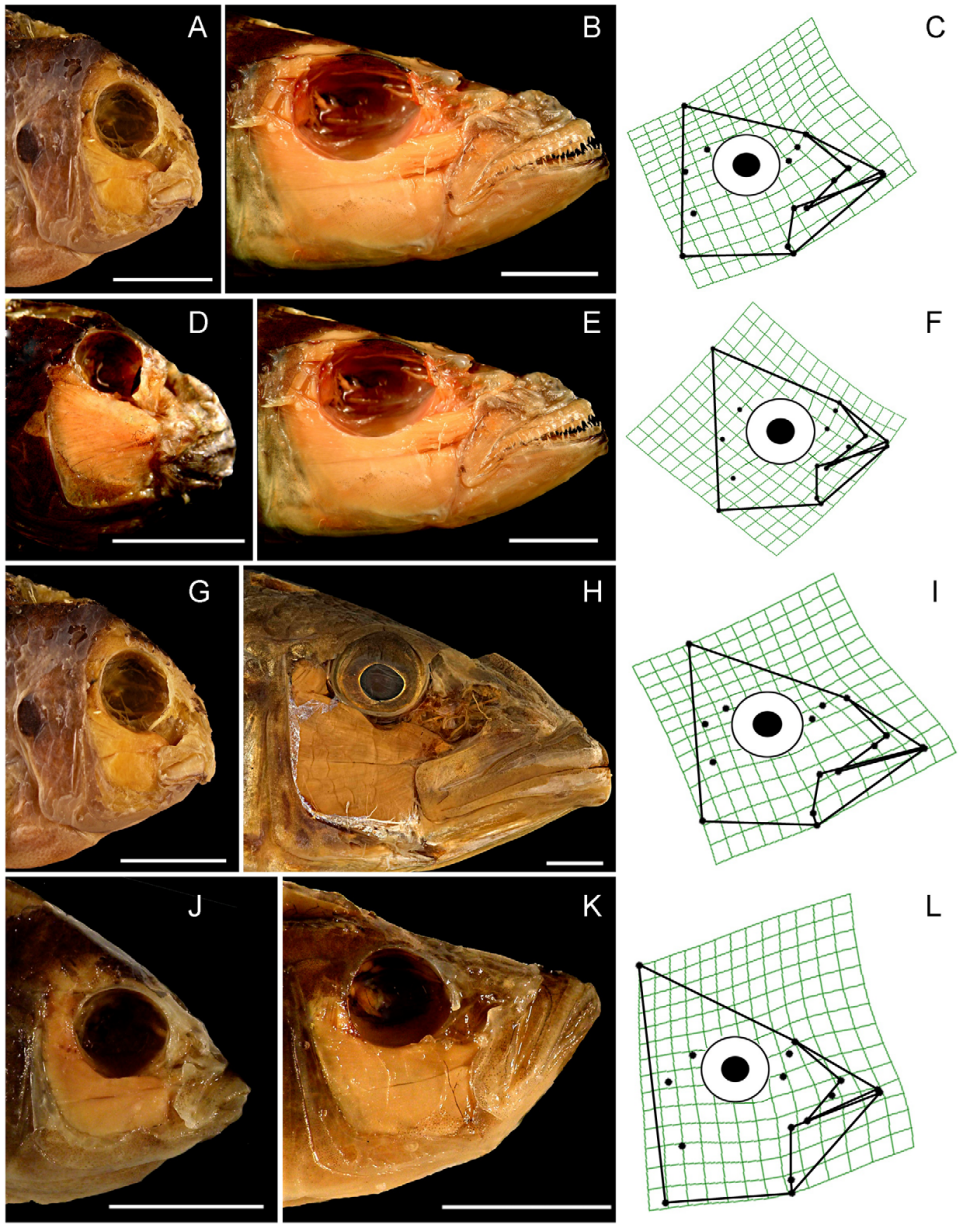
Pictorial descriptions of the shape variation described by PC1 in each of the four datasets. Plates A–L display pairwise comparisons of cichlids whose head shapes are strongly separated along PC1, but which are otherwise very similar (they have similar scores on other axes). The paired species from each dataset occupy the same row, and are immediately followed by a deformation grid that depicts the shape transformation associated with the PC1 axis in question. 1st row = All Lakes, 2nd row Lake Tanganyika, 3rd row = Lake Malawi, 4th row = Lake Victoria. A: *Labeotropheus fuelleborni*. B: *Bathybates fasciatus*. C: All Lakes PC1 deformation grid. D: *Spathodus* sp. E: *Bathybates fasciatus*. F: LT PC1 deformation grid. G: *Labeotropheus fuelleborni*. H. *Tyrannochromis macrostoma*. I: LM PC1 deformation grid. J: *Neochromis nigricans*. K: *Pyxichromis parorthostoma*. L: LV PC1 deformation grid. Scale bars = 1 cm.

**Figure 5:**
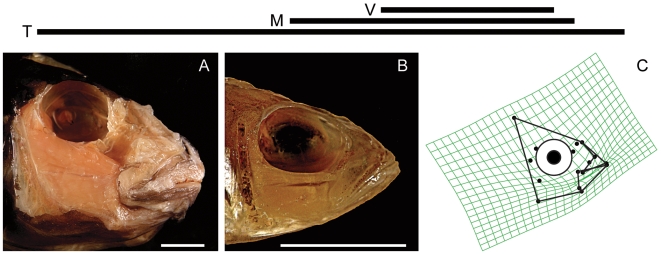
Pictorial description of the shape variation described by PC2 in the All Lakes dataset. The horizontal lines describe the relative extent of the distributions of the variation present in each lake along PC2. Top line = LV, Middle line = LM, Bottom line = LT. Plates A and B depict specimens whose head shapes are strongly separated along PC2, but which are otherwise very similar (they have similar scores on other axes). Only specimens from the All Lakes dataset are depicted. A. *Lobochilotes labiatus*. B. *Trematocara nigrifrons*. C: All Lakes PC2 deformation grid. Scale bars = 1 cm.

The similar orientations of PC1 and PC2 for all three lakes permit a straightforward comparison of the types of skull morphology that were most strongly distinguished by these axes (Figures 4 and 5). PC1 describes extreme differences in the size of the preorbital region of the skull, particularly jaw length (Figure 4), which is a character of great significance to trophic ecology [21], [33]–[35]. Even the fishes from LV, which is by far the youngest lake, display strong variation in the size of their upper and lower jaws relative to the size of the rest of the head (Figure 4).

In comparison to cichlids from LT, LM fishes exhibit only a subset of the shape variation described by PC2, while LV fishes occupy an even smaller portion of this range (Figures 3 and 5). This axis describes differences in eye size, the vertical placement of the eye, the distance between the eye and the mouth, the size of the jaw muscles, the relative height of the posterior edge of the skull, the orientation of the jaw, and the relative thickness of the jaw bones (Figure 5). This axis distinguishes smaller, gracile fishes with large eyes that should be capable of producing rapid bite sequences, from Tanganyikan benthic species that feed by producing hard bites, regardless of their preference for algae, animal prey, or a mixture of the two. Fishes from the Tanganyikan tribe Tropheini [36], which is the sister clade to the lineage that gave rise to the LM and LV radiations [1], are distinguished from most other fishes, including their close relatives in LM and LV, by this axis (Figure 3).

### Ecomorphological Groupings

The central region of the two-dimensional common cichlid shape space (Figure 3) is associated with an extremely wide range of trophic habits, and includes the morphologies of fishes that feed on various combinations of the following: filamentous algae and algal biocovers (*aufwuchs*); microorganisms that can be gleaned from *aufwuchs*; mollusks (including both snails and bivalves); benthic, pelagic and infaunal invertebrates; whole fishes; parts of fishes (e.g., fins, scales and spines); fish eggs and fish fry; and plankton [9], [10], [37]. Many of the species in this area of shape-space can be considered omnivorous. It is only at the periphery that we see strong associations between head morphology and specific sets of feeding habits (Figure 3).

Group A contains predatory fishes with long jaws and large gapes (Figure 3, Figure 4B), while group B is composed of smaller, more gracile fishes with large eyes placed close to upturned jaws (Figure 3, Figure 5B). Group C contains species from LM that feed on benthic food items that include arthropods, mollusks and algae, and all of these fishes are capable of producing strong bites (Figure 3, Figure 4A). Group D is composed of biting and “picking” fishes from LT that also consume benthic prey or algae, but which have taller heads, smaller eyes placed higher on the body, more robust jaws, and larger jaw muscles relative to the fishes in group C (Figure 3, Figure 5D). In groups C and D, the relative positions of the predators (*Cheilochromis euchilus*, 8; *Chilotilapia rhoadesii*, 9; *Chalinochromis brichardi*, 56; *Cyphotilapia frontosa*, 59; *Lobochilotes labiatus*, 72; *Spathodus* sp., 81) and the herbivores (*Labeotropheus fuelleborni*, 26; *Pseudotropheus* “red cheek”, 48, *Pseudosimochromis curvifrons*, 79; *Simochromis diagramma*, 80; *Tropheus brichardi*; 85) are maintained, with the herbivores consistently possessing shorter jaws and smaller preorbital regions (Figure 3). This difference will, all other factors being equal, permit a stronger bite in the herbivores [21], [33]–[35].

### Comparative Morphological Integration

**Figure 6:**
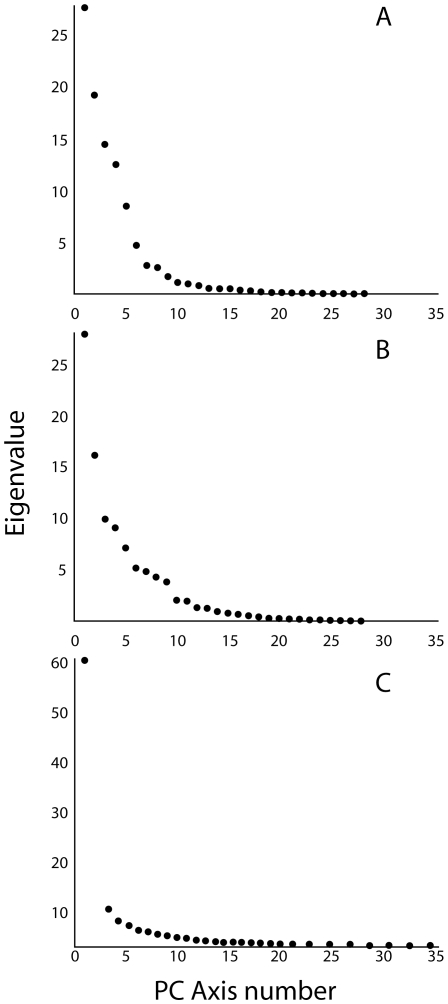
Scree plots of eigenvalues for the PC axes derived from PCAs of the individual lakes datasets. A = Lake Tanganyika. B = Lake Malawi. C = Lake Victoria.

The amount of morphological integration exhibited by the fishes from each lake was estimated by the distribution of eigenvalues derived from their respective PW matrices [38]–[41]. Inspection of the scree plots indicates that the total shape variation present among the specimens from each lake was distributed differently among their PC axes (Figure 6). Morphological variation was distributed most evenly among the axes that were calculated from the LT data, while data from the LV fishes were least uniform (Figure 6). This trend is best illustrated by differences in the relative contribution of PC1 to the overall variation, which was distinct in LV. A greater similarity between the PC1 and PC2 eigenvalues was observed for LT (27.74 and 19.25, respectively), compared to those for LM (28.09 and 16.23) and LV (60.84 and 11.69). These data indicate a high level of positional covariation among the anatomical landmarks of the LV fishes, i.e., their head morphology is highly integrated (Figure 6). The ranking of the relative levels of integration among the cichlid fishes from the three major East-African rift lakes is: LV>LM>LT (Figure 6).

## Discussion

The morphology of cichlid oral jaws has repeatedly diverged along similar trajectories in all three major East-African rift-lake radiations in ways that suggest predictable functional and ecological consequences related to bentho-pelagic feeding (Table 2; Figures 3, 4, 5) [11]. The cichlids in these lakes appear to be in different stages of a similar process of diversification such that the degree of anatomical diversity present within a lake is associated with the age of that lake (Tables 1 and 2; Figures 2–5). These anatomical changes are congruent with what would be expected if replicate adaptive radiations were examined at different points in time, and our results are therefore consistent with those of Young et al. [11], in that we observe what appear to be broadly repeated patterns of cichlid evolution.

The relative size of the preorbital region of the head, including the length of the oral jaws, accounts for the largest single type of morphological variation present in every lake (Table 2; Figures 3 and 4), and the extent of preorbital diversification has been prodigious in all three cases (Figure 4). PC1 scores for those fishes from the two oldest lakes (LT, 8–16 MYA; LM, 2–4 MYA) have similar ranges along this axis, even though the maximum divergence time for LM cichlids has been considerably less (Table 3; Figure 3) [17]. Even the cichlids endemic to LV have expanded into more than 50% of this distribution in an extremely brief period of evolutionary time (0.015–0.2 my; Figure 3) [17]. It is also notable that preorbital size evolution is the only type of craniofacial divergence that has progressed to any appreciable degree among the LV cichlids (>60% of the total skull shape variation).

### Novel Head Shapes among the Tropheini

An interesting pattern emerges when comparing the head shapes of cichlids from LM and LV to their closest Tanganyikan relatives: the tribe Tropheini [1], [3]. These three lineages represent the majority of the “modern haplochromines”, a monophyletic branch of the Cichlidae that also includes some East-African riverine cichlids, and whose common ancestor lived approximately 1.8 MYA [1]. A reasonable assumption would be that due to their close relationship the modern haplochromines should display a wide degree of overlap in head morphology. While this pattern is clearly evident in the case of the LM and LV clades (Table 3; Figure 3), the Tropheini occupy a region of head shape space that is almost entirely peripheral to the area defined by these close relatives (Figure 3). A possible explanation for this pattern is that, whereas the ancestors of LM and LV haplochromines encountered few, if any, resident cichlid or other perciform fish populations as they populated these lakes, the Tropheini invaded (or re-invaded) a lake in which cichlids had already diversified extensively over several million years [17]. Competition with established cichlids might therefore have pressured the Tropheini to diversify in a less densely occupied region of shape space (i.e., character displacement; Figure 3).

### Bentho-Pelagic Divergence among Fishes

The extremes of the 1st PC axis are occupied by benthic and pelagic feeding fishes in every analysis (Figure 3), and this trend exhibits striking similarity to patterns of bentho-pelagic divergence that have evolved rapidly among a wide range of fish species, including cichlids, sunfishes, sticklebacks, whitefishes, perch, and arctic charr [42]–[66]. In numerous cases this ecological differentiation has been shown to be associated with divergence in jaw/head length similar to that described by PC1 [54], [55], [57]–[60], [62]–[68]. The large amount of evidence from fishes that have recently invaded post-glacial lakes (in both hemispheres) is particularly pertinent, since it indicates that the rapid diversification of jaw length has repeatedly occurred almost immediately when fishes have invaded a newly formed lake, and the majority of these studies link such changes to divergence along a bentho-pelagic feeding axis [48], [54], [57], [63]–[65], [69]–[71]. Among threespine sticklebacks and arctic charr, assortative mating is thought to have played an important role in the transition from anatomically distinct benthic/pelagic morphs to reproductively isolated species [52], [72]–[77], providing a potential mechanism for rapid speciation along this ecomorphological axis.

There is also evidence that bentho-pelagic divergence in trophic morphology can appear within the first generation of fishes that invade a new lake. Multiple studies of phenotypic plasticity in several distantly related teleost fishes have shown that restriction to either a benthic or pelagic diet during development will produce adult head morphologies that are similar to those of species that specialize on such diets [50], [54], [60], [61], [67], [68], [78]–[80]. Of particular interest are studies that have demonstrated such plasticity in cichlids from both LM and LV [60], [80]. It appears highly plausible that developmental plasticity in the trophic morphology of cichlid oral jaws could represent an ancestral “flexible stem” that promoted their rapid and repeated evolutionary divergence along a common bentho-pelagic feeding axis [50], [81], and there are strong indications that this could be a general phenomenon when fishes invade new lakes.

Whether lakes Malawi and Tanganyika went through an initial period of explosive preorbital evolution similar to what LV has experienced is unknown. Although our data are consistent with such a pattern, they do not permit a thorough test of this hypothesis. However, the combined evidence from the African rift-lake cichlids, fishes that have recently invaded post-glacial lakes, and studies of rapid bentho-pelagic divergence in a large number of other fish taxa, strongly indicates that preorbital size divergence is likely to occur with great rapidity when fishes invade new environments with multiple open niches. An ability to rapidly evolve jaws of different lengths (i.e., different preorbital sizes), and an associated ability to undergo an evolutionary transition between benthic and pelagic feeding niches, has been documented in the marine damselfishes [21], which are close relatives to the cichlids [82]–[85].

The evolutionary advantages of being able to rapidly change jaw size are extensive, since such adjustments will produce substantial differences in the biomechanics of fish feeding that will result in important shifts in trophic ecology [21], [33]–[35], [86]–[90]. Fishes with short jaws have the potential to produce bites that are proportionally more powerful due to the increased mechanical advantage that would be applied by similarly sized jaw muscles. Such attributes are advantageous for herbivores that either bite off pieces of plant material or scrape algae from the substrate, and for benthic predators that generate larger bite forces in order to subdue, crush, puncture, sever, dismember, detach, or uncover their prey [21], [33]–[35]. Longer jaws enable the capture of larger prey, increase the speed of the jaws during biting, and can promote greater jaw protrusion, all of which have been repeatedly linked to an enhanced ability to capture elusive prey in multiple perciform lineages [34], [87], [88], [91]–[95]. The rapid evolution of changes in preorbital size therefore promotes the adaptive radiation of trophic morphology via functional shifts in the biomechanics of fish skulls.

### The Preorbital Region As an Evolutionary Module

A module is a unit that is tightly integrated internally, but which is relatively independent from other such modules [23], [96]. We suggest that the bones of the preorbital region (e.g., the articular, retro-articular, dentary, maxilla, premaxilla, nasal, and palantine), all of which are directly associated with jaw functioning, are likely to constitute an anatomical, functional and evolutionary module among the rift-lake cichlids. The largest aspect of head shape diversity was variation in preorbital size, and this variation was described by a single, shared PC trajectory across all datasets (Figures 3 and 4, Table 3). Because of how a PCA is calculated, the shape variation described by a single axis is statistically independent of the other types of morphological variation present in the data, and such strongly repeated patterns suggest the presence of an anatomical module. The size and shape of the bones in this region also have strong functional consequences for fish trophic biomechanics, and these structures must operate together in order for successful feeding to occur [21], [35], [86], [91], [97]–[105]. Furthermore, the evolvability of preorbital size is underscored by the finding that such changes accounted for the most pronounced aspects of anatomical variation within all three cichlid radiations (Figures 3 and 4, Table 3). Even in the extremely young LV species flock, preorbital evolution has been extensive (Figure 4) while other types of cranial anatomical diversification have yet to progress much at all (Figures 3 and 6).

Modularity of the preorbital region of the teleost skull is further supported by the study of zebrafish mutants. At least three different mutants have been characterized that exhibit discrete oral jaw shortening [106]–[108]. Two of these are deficient in Fgf ligands [107], [108], and the other lacks Glypican 4, a member of the glypican (GPC) family of extracellular proteins, which exert their effects over development by modulating Wnt, Bmp, and Fgf signaling [106]. The similarity of these mutational effects in regard to craniofacial geometry suggests a common developmental mechanism. The observation that single genetic lesions can result in pronounced variation in oral jaw length suggests that homologous variation among cichlid species may have a similarly tractable genetic basis.

The shape changes described by PC2 (Figures 3 and 5) are also consistent among the three groups of cichlids, but in this case we observe stronger differences in the extent of within-lake shape variation, and the these patterns are consistent with the evolution of these traits proceeding more slowly than changes in preorbital size (Figures 3 and 5). These anatomical shifts involve changes in both the anterior and posterior regions of the head, and given the disparate functions performed by the posterior region of the skull, which include feeding, respiration and vision, it is likely that it is composed of multiple anatomical modules. If this prediction is true, then coordinated changes between the two regions would be expected to proceed more slowly than changes that are restricted to single modules. Furthermore, the changes in eye size, eye position, jaw bone thickness and skull height that are associated with this axis are likely to be currently undergoing divergence in the two youngest lakes (Figures 2 and 5), and the differences between the “hard-biting” fishes in LT and LM (i.e., Figure 3, groups C and D) suggest that not all ecological niches have been fully exploited in the younger lakes.

### Conclusions

We present strong evidence that cichlid trophic radiations have followed similar patterns of divergence in the Great East-African rift lakes. The primary axis of anatomical variation among rift-lake cichlids is associated with changes in the preorbital region that are largely independent from the rest of the skull, and such changes are associated with biomechanical shifts in jaw function that distinguish benthic-feeding fishes from pelagic-feeding fishes. We suggest that this type of divergence may proceed rapidly during the early stages of the ecomorphological diversification that occurs when fishes colonize new environments with many open niches.

It is probable that the preorbital region of the head constitutes a distinct developmental and evolutionary module in cichlids, and possibly other groups as well. The recent finding of very low levels of genetic diversity among Lake Malawi cichlids [109] suggests that this radiation has occurred in the context of genomic uniformity, which makes it likely that selection has targeted alleles with major effects on discrete functional modules. An emerging group of literature also indicates that a small number of genetic changes of large effect often accompany rapid adaptive radiations [110]–[121]. These finding raise the hypothesis that cichlid preorbital size may be controlled by relatively few genes of large effect, a theory that is supported by genetic work in the zebrafish [106]–[108]. Testing this hypothesis will contribute to our understanding of the evolution of the rift-lake cichlids specifically, and of the proximate mechanisms that have directed adaptive radiations in fish feeding in general.

## Supporting Information

Table S1 Species list with specimen numbers.
(0.12 MB DOC)

Table S2 Supplementary key for Figure 2.
(0.08 MB DOC)
