# Supplementary material for: Bentho-Pelagic Divergence of Cichlid Feeding Architecture Was Prodigious and Consistent during Multiple Adaptive Radiations within African Rift-Lakes
Source: PLoS One. 2010 Mar 8;5(3):e9551. doi: 10.1371/journal.pone.0009551 (PMC2833203; doi:10.1371/journal.pone.0009551)
Supplement: Table S1 — Species list with specimen numbers. (0.12 MB DOC) [file pone.0009551.s001.doc]

Species list with specimen numbers.*

| Lake Tanganyika | Specimen numbers |
| --- | --- |
| *Altolamprologus compressiceps (*3) | CU 88649 |
| *Aulonocranus dewindti* (3) | MCZ 32624 |
| *Bathybates fasciatus* (3) | CU 88627 |
| *Callochromis pleurospilus* (3) | MCZ 50826 |
| *Cardiopharynx schoutedeni* (3) | MCZ 50827 |
| *Chalinochromis brichardi* (2) | CU 88628 |
| *Cyathopharynx furcifer* 1 (3) | CU 89305 |
| *Cyathopharynx furcifer* 2 (3) | MCZ 50828 |
| *Cyphotilapia frontosa* (3) | CU 88626 |
| *Cyprichromis leptosoma* (3) | CU 88630 |
| *Ectodus descampsii* (3) | MCZ 49282 |
| *Gnathochromis pfefferi* (2) | CU 88652 |
| *Grammatotria lemairii* (3) | MCZ 49277 |
| *Haplotaxodon microlepis* (1) | CU 88641 |
| *Hemibates stenosoma* (3) | MCZ 50829 |
| *Julidochromis marlieri* (3) | CU 89307 |
| *Lamprologus callipterus* (3) | CU 88650 |
| *Lepidiolamprologus elongatus* (2) | CU 9077 |
| *Lestradea stappersii* (3) | MCZ 32593 |
| *Limnochromis auritus* (1) | MCZ 49271 |
| *Limnotilapia dardennii* (2) | CU 88642 |
| *Lobochilotes labiatus* (3) | CU 88643 |
| *Neolamprologus furcifer* (3) | CU 88629 |
| *Ophthalmotilapia ventralis* (3) | CU 88644 |
| *Perissodus microlepis* (long side) (1) | MRAC 94-69-p-0673-685 |
| *Perissodus microlepis* (short side) (1) | MRAC 94-69-p-0673-685 |
| *Plecodus straeleni* (long side) (2) | UMMZ 199781 |
| *Plecodus straeleni* (short side) (2) | UMMZ 199781 |
| *Pseudosimochromis curvifrons* (2) | CU 89316 |
| *Simochromis diagramma* (2) | CU 88719 |
| *Spathodus* sp*.* (2) | CU 89317 |
| *Telmatochromis temporalis* (1) | CU 88633 |
| *Trematocara nigrifrons* (3) | MCZ 50702 |
| *Triglachromis otostigma* (1) | MCZ 49275 |
| *Tropheus brichardi* (2) | CU 93700 |
| *Tylochromis lateralis* (2) | CU 91570 |
| *Xenochromis hecqui* (2) | MCZ 49334 |
| *Xenotilapia* sp. (1) | CU 82933 |
| Lake Malawi | Specimen numbers |
| *Alticorpus profundicola* (3) | MRAC 99-041-P-3747-3749 |
| *Aristochromis christyi* (1) | MCZ 49522 |
| *Astatotilapia calliptera* (2) | AMNH 97330 |
| *Buccochromis atritaeniatus* (1) | MCZ 49460 |
| *Caprichromis orthognathus* *(*2) | AMNH 225477 |
| *Champsochromis spilorhynchus* (1) | MCZ 49454 |
| *Cheilochromis euchilus* (2) | MRAC 79-040-P-16-17 |
| *Chilotilapia rhoadesii* (4) | Aquarium trade specimens |
| *Copadichromis borleyi* (3) | Wild-caught, aquarium trade specimens |
| *Copadichromis virginalis* (3) | AMNH 31858 |
| *Corematodus taeniatus* (1) | AMNH 225469 |
| *Ctenopharynx pictus* (1) | AMNH 31788 |
| *Cyathochromis obliquidens* (1) | MCZ 49440 |
| *Cynotilapia afra* (1) | Wild-caught, aquarium trade specimens |
| *Cyrtocara moorii* (1) | MCZ 96429 |
| *Dimidiochromis compressiceps* (3) | Wild-caught, aquarium trade specimens |
| *Diplotaxodon argenteus* (1) | MCZ 135962 |
| *Docimodus johnstoni* (1) | MCZ 49526 |
| *Exochochromis anagenys* (2) | Wild-caught, aquarium trade specimens |
| *Fossorochromis rostratus* (2) | MRAC 99-041-P-5164-5165 |
| *Genyochromis mento* (1) | AMNH 223496 |
| *Gephyrochromis lawsi* (3) | MRAC 99-041-P-2021-2023 |
| *Hemitaeniochromis urotaenia* (1) | MCZ 49539 |
| *Hemitilapia oxyrhyncha* (2) | AMNH 225466 |
| *Iodotropheus sprengerae* (3) | MRAC 91-054-P-36-37 |
| *Labeotropheus fuelleborni* (3) | Wild-caught, aquarium trade specimens |
| *Labidochromis caeruleus* (2) | Aquarium trade specimens |
| *Maylandia* (*Metriaclima*) *zebra (*6) | Wild-caught, aquarium trade specimens |
| *Melanochromis auratus* (2) | Wild-caught, aquarium trade specimens |
| *Mylochromis sphaerodon* (1) | MCZ 49516 |
| *Nimbochromis linni* (3) | Wild-caught, aquarium trade specimens |
| *Nimbochromis livingstonii* (1) | AMNH 221782 |
| *Nyassachromis leuciscus* (1) | MCZ 49517 |
| *Otopharynx heterodon* (2) | AMNH 31762 |
| *Otopharynx lithobates* (1) | Wild-caught, aquarium trade specimens |
| *Pallidochromis tokolosh* (3) | MRAC 99-041-P-8105, MRAC 99-041-P-866-867 |
| *Petrotilapia* sp. (1) | MCZ 60445 |
| *Placidochromis subocularis* (2) | AMNH 221766 |
| *Protomelas* "bluefire" (2) | Wild-caught, aquarium trade specimens |
| *Pseudotropheus*  (*Tropheops*) "red fin" (3) | Wild-caught, aquarium trade specimens |
| *Pseudotropheus* (*Tropheops*) "red cheek" (4) | Wild-caught, aquarium trade specimens |
| *Pseudotropheus tropheops* (2) | AMNY 927-95 |
| Lake Malawi cont. | Specimen numbers |
| *Rhamphochromis macrophthalmus* (1) | AMNH 92604 |
| *Sciaenochromis ahli* (2) | AMNH 225452 |
| *Stigmatochromis pleurospilus* (3) | AMNH 224671 |
| *Taeniochromis holotaenia* (1) | AMNH 221751 |
| *Taeniolethrinops praeorbitalis* (2) | AMNH 225480 |
| *Tramitichromis brevis* (1) | MCZ 49452 |
| *Trematocranus placodon* (2) | AMNH 98196 |
| *Tyrannochromis macrostoma* (1) | MCZ 49534 |
|  |  |
|  |  |
| Lake Victoria |  |
| *Astatoreochromis alluaudi* (2) | MCZ 100143, MCZ 100161 |
| *Haplochromis guiarti* (3) | MCZ 60296 |
| *Haplochromis lividus* (1) | MCZ 100654 |
| *Haplochromis phytophagus* (1) | MCZ 100227 as *Xystichromis phytophagus* |
| *Haplochromis* *retrodens* (1) | MCZ 60308 as *Hoplotilapia retrodens* |
| *Haplochromis rubescens* (2) | MCZ 153512 as *Haplochromis ruby* |
| *Macropleurodus bicolor* (3) | MCZ 100579 |
| *Mbipia mbipi* 3 | MCZ 100160 |
| *Neochromis nigricans* (3) | MCZ 131294 |
| *Paralabidochromis* "rock-kribensis" (1) | MCZ 100145 |
| *Pseudocrenilabrus multicolor* (3) | MCZ 155324 |
| *Pyxichromis parorthostoma* (3) | MCZ 153518 as *Pyxichromis orthostoma* |

*The number of specimens examined and select junior synonyms in parentheses. Undescribed species in quotations. American Museum of Natural History (AMNH). Belgian Royal Museum for Central Africa (MRAC). Cornell University's Museum of Vertebrates (CU). Museum of Comparative Zoology, Harvard (MCZ). The University of Michigan's Museum of Zoology (UMMZ)
