# Supplementary material for: Bentho-Pelagic Divergence of Cichlid Feeding Architecture Was Prodigious and Consistent during Multiple Adaptive Radiations within African Rift-Lakes
Source: PLoS One. 2010 Mar 8;5(3):e9551. doi: 10.1371/journal.pone.0009551 (PMC2833203; doi:10.1371/journal.pone.0009551)
Supplement: Table S2 — Supplementary key for Figure 2. (0.08 MB DOC) [file pone.0009551.s002.doc]

Supplementary key for Figure 2.

|  | Lake Malawi species (1-50) |  | Lake Tanganyika species (51-88) |
| --- | --- | --- | --- |
| 1 | *Alticorpus profundicola* | 51 | *Altolamprologus compressiceps* |
| 2 | *Aristochromis christyi* | 52 | *Aulonocranus dewindti* |
| 3 | *Astatotilapia calliptera* | 53 | *Bathybates fasciatus* |
| 4 | *Buccochromis atritaeniatus* | 54 | *Callochromis pleurospilus* |
| 5 | *Cynotilapia afra* | 55 | *Cardiopharynx schoutedeni* |
| 6 | *Caprichromis orthognathus* | 56 | *Chalinochromis brichardi* |
| 7 | *Champsochromis spilorhynchus* | 57 | *Cyathopharynx furcifer* 1 |
| 8 | *Cheilochromis euchilus* | 58 | *Cyathopharynx furcifer* 2 |
| 9 | *Chilotilapia rhoadesii* | 59 | *Cyphotilapia frontosa* |
| 10 | *Copadichromis borleyi* | 60 | *Cyprichromis leptosoma* |
| 11 | *Copadichromis virginalis* | 61 | *Ectodus descampsii* |
| 12 | *Corematodus taeniatus* | 62 | *Gnathochromis pfefferi* |
| 13 | *Ctenopharynx pictus* | 63 | *Grammatotria lemairii* |
| 14 | *Cyathochromis obliquidens* | 64 | *Haplotaxodon microlepis* |
| 15 | *Cyrtocara moorii* | 65 | *Hemibates stenosoma* |
| 16 | *Dimidiochromis compressiceps* | 66 | *Julidochromis marlieri* |
| 17 | *Diplotaxodon argenteus* | 67 | *Lamprologus callipterus* |
| 18 | *Docimodus johnstoni* | 68 | *Lepidiolamprologus elongatus* |
| 19 | *Exochochromis anagenys* | 69 | *Lestradea stappersii* |
| 20 | *Fossorochromis rostratus* | 70 | *Limnochromis auritus* |
| 21 | *Genyochromis mento* | 71 | *Limnotilapia dardennii* |
| 22 | *Gephyrochromis lawsi* | 72 | *Lobochilotes labiatus* |
| 23 | *Hemitaeniochromis urotaenia* | 73 | *Neolamprologus furcifer* |
| 24 | *Hemitilapia oxyrhyncha* | 74 | *Ophthalmotilapia ventralis* |
| 25 | *Iodotropheus sprengerae* | 75 | *Perissodus microlepis* (short side) |
| 26 | *Labeotropheus fuelleborni* | 76 | *Perissodus microlepis* (long side) |
| 27 | *Labidochromis caeruleus* | 77 | *Plecodus straeleni* (short side) |
| 28 | *Mylochromis sphaerodon* | 78 | *Plecodus straeleni* (long side) |
| 29 | *Melanochromis auratus* | 79 | *Pseudosimochromis curvifrons* |
| 30 | *Maylandia* (*metriaclima*) *zebra* | 80 | *Simochromis diagramma* |
| 31 | *Nimbochromis linni* | 81 | *Spathodus* sp, |
| 32 | *Nimbochromis livingstonii* | 82 | *Telmatochromis temporalis* |
| 33 | *Nyassachromis leuciscus* | 83 | *Trematocara nigrifrons* |
| 34 | *Otopharynx heterodon* | 84 | *Triglachromis otostigma* |
| 35 | *Otopharynx lithobates* | 85 | *Tropheus brichardi* |
| 36 | *Pallidochromis tokolosh* | 86 | *Tylochromis lateralis* |
| 37 | *Petrotilapia* sp. | 87 | *Xenochromis hecqui* |
| 38 | *Placidochromis subocularis* | 88 | *Xenotilapia* sp. |
| 39 | *Protomelas* "bluefire" |  | Lake Victoria species (89-100) |
| 40 | *Pseudotropheus tropheops* | 89 | *Astatoreochromis alluaudi* |
| 41 | *Rhamphochromis macrophthalmus* | 90 | *Haplochromis guiarti* |
| 42 | *Sciaenochromis ahli* | 91 | *Haplochromis lividus* |
| 43 | *Stigmatochromis pleurospilus* | 92 | *Haplochromis rubescens* |
| 44 | *Taeniochromis holotaenia* | 93 | *Haplochromis retrodens* (*Hoplotilapia*) |
| 45 | *Taeniolethrinops praeorbitalis* | 94 | *Macropleurodus bicolor* |
| 46 | *Tramitichromis brevis* | 95 | *Mbipia mbipi* |
| 47 | *Trematocranus placodon* | 96 | *Neochromis nigricans* |
| 48 | *Pseudotropheus* (*Tropheops*) "red cheek" | 97 | *Paralabidochromis* "rock-kribensis" |
| 49 | *Pseudotropheus* (*Tropheops*) "red fin" | 98 | *Pseudocrenilabrus multicolor* |
| 50 | *Tyrannochromis macrostoma* | 99 | *Haplochromis phytophagus* (*Xystichromis*) |
|  |  | 100 | *Pyxichromis parorthostoma* |
